# Women with exposure to childhood interpersonal violence without psychiatric diagnoses show no signs of impairment in general functioning, quality of life and sexuality

**DOI:** 10.1186/s40479-016-0048-y

**Published:** 2016-10-07

**Authors:** Sophie Rausch, Julia Herzog, Janine Thome, Petra Ludäscher, Meike Müller-Engelmann, Regina Steil, Kathlen Priebe, Thomas Fydrich, Nikolaus Kleindienst

**Affiliations:** 1 Institute of Psychiatric and Psychosomatic Psychotherapy, Central Institute of Mental Health Mannheim, Medical Faculty Mannheim, Heidelberg University, J5, 68159 Mannheim, Germany; 2 Department of Psychosomatic Medicine and Psychotherapy, Central Institute of Mental Health Mannheim, Medical Faculty Mannheim, Heidelberg University, J5, 68159 Mannheim, Germany; 3 Department of Clinical Psychology and Intervention, Institute of Psychology, Goethe University Frankfurt, Varrentrappstr. 40-42, 60486 Frankfurt am Main, Germany; 4 Department of Psychology, Faculty of Life Sciences, Humboldt-Universitaet zu Berlin, Unter den, Linden 6, 10999 Berlin Germany

**Keywords:** Childhood sexual abuse, Childhood physical abuse, Psychopathology, Quality of life, Sexuality

## Abstract

**Background:**

Childhood interpersonal violence is a major risk factor for developing Posttraumatic Stress Disorder (PTSD), other axis-I disorders or Borderline Personality Disorder (BPD). Individuals with a history of childhood sexual abuse (CSA) and childhood physical abuse (CPA) who meet the criteria of any axis-I disorder usually also exhibit general psychopathologic symptoms and impairments in quality of life and sexuality. The present study investigates whether women with a history of potentially traumatic CSA/CPA without any axis-I disorder or BPD show subthreshold symptoms of PTSD-specific and general psychopathology and impairments in global functioning, quality of life, and sexuality.

**Methods:**

Data were obtained from *N* = 92 female participants: *n* = 31 participants with a history of potentially traumatic CSA/CPA (defined as fulfilling PTSD criterion A) without any axis-I disorder or BPD; *n* = 31 participants with PTSD related to CSA/CPA; and *n* = 30 healthy controls without any traumatic experiences. All three groups were matched for age and education. Those with a history of CSA/CPA with and without PTSD were further matched with regard to severity of physical and sexual abuse.

**Results:**

While women with a history of potentially traumatic CSA/CPA without axis-I disorder or BPD clearly differed from the PTSD-group in the collected measures, they did not differ from healthy controls (e.g., GAF:87, BSI:0.3, BDI-II:4.5). They showed neither PTSD-specific nor general subthreshold symptoms nor any measurable restrictions in quality of life or sexual satisfaction.

**Conclusions:**

Women with a history of potentially traumatic childhood interpersonal violence without axis-I disorder or BPD show a high level of functioning and a low level of pathological impairment that are comparable to the level of healthy controls. Further studies are needed to identify what helped these women survive these potentially traumatic experiences without developing any mental disorders.

**Trial registration:**

German Clinical Trials Registration ID: DRKS00006095. Registered 21 May 2014.

## Background

The quest for protective factors that prevent people from developing diagnosable psychopathology in the aftermath of potentially traumatic events (PTE) has recently gained much attention in research. The term “PTE” subsumes different events such as natural disasters, motor vehicle accidents, serious injury, or childhood interpersonal violence [1]. Among those affected by PTE, only a minority develops Posttraumatic Stress Disorder (PTSD). Some develop partial PTSD (high symptom levels that do not meet full diagnostic criteria [2]), and some do not develop any manifest axis-I or axis-II disorder. Previous studies have examined the epidemiology of psychopathology (e.g., PTSD) as a reaction to PTE. A large national comorbidity survey in the U.S. estimated the conditional probabilities of developing PTSD after PTE to be around 20.4 % in women (lifetime prevalence rate [3]). Maercker et al. have found conditional probabilities of developing PTSD after PTE in 12.0 % and of developing partial PTSD in another 12.8 % of male and female participants in a German representative epidemiologic study [4]. Estimates of PTSD have varied depending on the different types of traumatic events. In a study by Ehlers et al., 16.5 % of survivors of motor accidents developed PTSD, but the vast majority did not meet the criteria, with subjects showing only 3.3 PTSD symptoms on average [5]. When looking at incidence of PTSD-diagnoses in veterans, 12.5 % of the examined Gulf War veterans developed PTSD and 25 % suffered from partial PTSD, while 62.5 % had no psychological impairments 1 year after returning to the United States [6]. A similar pattern was found for Manhattan residents who survived the 9/11 terrorist attacks in New York: 7.5 % developed PTSD, 17.4 % developed partial PTSD, and the rest did not report a single PTSD-symptom [7]. However, when experiences of childhood interpersonal violence are considered, the rates of subsequent PTSD are substantially higher, with estimates of PTSD in childhood or adolescence ranging between 20 and 90 % [8] and increased odds ratios for developing other axis-I disorders in childhood or adulthood (Affective Disorders OR: 1.75–3.57; Anxiety Disorders: OR: 1.69–3.21; Substance Abuse Disorders OR: 1.0–4.14) [3, 9–14]. In a large U.S. [3], 39.1 % of women with a history of childhood sexual abuse (CSA) developed PTSD. In line with these numbers, German epidemiologic studies have found the conditional probability of developing PTSD after CSA in women ranges between 28.8 [15] and 35.3 % [4].

Aside from PTSD, psychopathological impairments after experiences of childhood interpersonal violence cover a broad range of symptomatology. A few prospective longitudinal studies have examined the impact of CSA or CPA on the level of functioning or help-seeking behavior in public mental health services. With regard to the level of functioning, Bolger and Patterson assessed 107 abused children in a prospective longitudinal study over a period of 5 years. Of those affected by childhood interpersonal violence, fewer than 5 % were functioning well over the study period of 5 years (from grade 2 until grade 5 [16]). With regard to help-seeking behaviors, Cutajar et al. followed 2759 registered CSA cases for a period of 43 years in Australia [9]. Of those 2759 affected by CSA, only 23.3 % had a lifetime record of using public mental health services. This number is surprisingly small. However, it has to be kept in mind that this study only captured contacts with public mental health services and did not register contacts with private health services, counseling groups or support groups. The study also did not cover the estimated number of unknown cases with mental health impairments who did not contact mental health services.

Those who developed PTSD in the aftermath of CSA not only show the hallmark symptoms of PTSD such as intrusions, avoidance, numbing and hyperarousal, but usually also a range of further symptoms. Previous cross-sectional studies with participants with a history of CSA with PTSD have found significantly higher depression scores, lower self-esteem, higher general psychopathology, more dissociative symptoms, more intense guilt cognitions and more impulsiveness compared to their non-abused counterparts [11, 17–23]. Additionally, experiences of childhood physical abuse (CPA) and CSA are often associated with reduced quality of life (QoL) [24, 25]. Previous studies have identified further risk factors for poorer QoL such as more pronounced PTSD and depressive symptomatology [26, 27].

However, psychopathological impairments and reduced QoL are not limited to those who meet full criteria of PTSD. People suffering from partial PTSD may as well have clinically significant symptoms that affect their mental and physical health and social relationships. Previous studies suggest that a diagnostic approach with a binary classification into present or absent PTSD diagnosis might not be sufficient to describe the impact of impairment in survivors of traumatic events. For example Stein et al. [2] found that survivors of PTE (e.g., rape, physical abuse, combat, natural disaster, etc.) show serious functional impairment even when full PTSD diagnostic criteria are not met. Although survivors with full PTSD reported significantly more impairment in work or school functioning than persons with partial PTSD, the latter still reported more impairment in work or school functioning than traumatized persons with fewer PTSD symptoms and non-traumatized persons. In terms of impaired home and social functioning, survivors with full and partial PTSD did not differ, but both groups experienced more impairment than survivors without PTSD and non-traumatized individuals. This subthreshold symptomatology is particularly common in participants with a history of CSA [28]. However, it has to be kept in mind that none of these studies controlled for co-occurring diagnoses. Therefore, the results could have been affected by ramifications of other psychopathologies such as depression or anxiety disorders. Furthermore, Stein et al.’s study provides only indirect evidence when examining the influence of childhood interpersonal violence because it included survivors of all kind of traumatic experiences. To our knowledge, no study has assessed partial PTSD in a sample of participants who had solely been affected by CSA/CPA.

Besides general psychopathological impairments, those affected by CSA frequently reported difficulties concerning their sexual relationships in adulthood [29]. Laumann et al. [30] examined the prevalence of adult sexual dissatisfaction and disturbances in CSA victims. Among those affected by CSA, 40 % lacked interest in sex, and 32 % reported that sex was not pleasurable. Several other studies examining victims of CSA replicated these findings [18, 31, 32]. However, none of these studies on sexual satisfaction assessed PTSD or other axis-I diagnoses and therefore did not distinguish between participants with and without psychopathology.

Besides studies on psychopathological impairments in participants with a history of CSA/CPA with full and partial PTSD, few studies have examined participants with a history of CSA/CPA *without* PTSD. Kleim et al. [33] showed that trauma survivors without PTSD experience only marginally less intrusions than those with PTSD, but they experience them less vividly. Concerning dissociation, Lanius et al. [34] showed that trauma survivors without PTSD experience significantly less dissociative symptoms as compared to trauma survivors with PTSD under experimentally induced distress in a laboratory setting. But these studies by Lanius and Kleim examined trauma survivors of all kinds of traumatic events (e.g., combat, assault, motor vehicle accidents, or CSA) and not only those affected by childhood interpersonal violence. Therefore, these results provide only indirect evidence of the influence of childhood interpersonal violence on dissociation and intrusive re-experiencing. In addition, all these studies on trauma survivors without PTSD included survivors who possibly met other axis-I or axis-II diagnoses. Thus, effects of pathologies other than PTSD were not considered.

In sum, previous research has shown that participants with a history of childhood interpersonal violence with full and partial PTSD show significant impairments in general functioning, quality of life, and sexuality. Research on trauma survivors without PTSD is scant and has significant limitations. Limitations emerge from the fact that different groups are only distinguished by means of the dichotomy into presence or absence of PTSD-diagnosis, but axis-I or axis-II disorders other than PTSD are not considered. Additionally, previous research has not attempted to minimize the impact of differences in severity of experienced CSA or CPA in those with and without PTSD when examining psychopathologic responses to traumatic events, which poses a threat to the internal validity of the results. To our knowledge, all these studies included participants without PTSD who potentially met other axis-I diagnoses. Hence, it remains unclear whether participants with no axis-I or axis-II disorders plus no experiences of psychotherapeutic interventions or intake of psychotropic medication with a history of potentially traumatic CSA/CPA suffer from subthreshold symptoms and exhibit at least unspecific restrictions in psychological well-being.

The present study examines whether healthy participants with a history of potentially traumatic CSA or CPA without any axis-I disorder or Borderline Personality Disorder (BPD;healthy trauma-exposed women; HTEW) differ from 1) healthy controls (HC) who have never experienced a trauma nor meet the diagnosis of a mental disorder and 2) participants with PTSD related to experiences of CSA/CPA (PTSD patients), in the following domains: dissociation, depression, global functioning, impulsivity, self-esteem, PTSD-specific psychopathology, satisfaction with mental and physical aspects of quality of life and sexuality. Furthermore, we were interested in current resilience scores of those three groups, as measured with a widely used resilience scale (RS; [35]). To avoid the potentially confounding influences of severity effects of CSA or CPA in individuals in the HTEW and PTSD groups, we controlled for severity by matching the individuals of the HTEW and the PTSD patient group with regard to their Childhood Trauma Questionnaire (CTQ; [36]) scores for the subscales of “Physical Abuse” and “Sexual Abuse”.

## Methods

### Sample

**Fig. 1:**
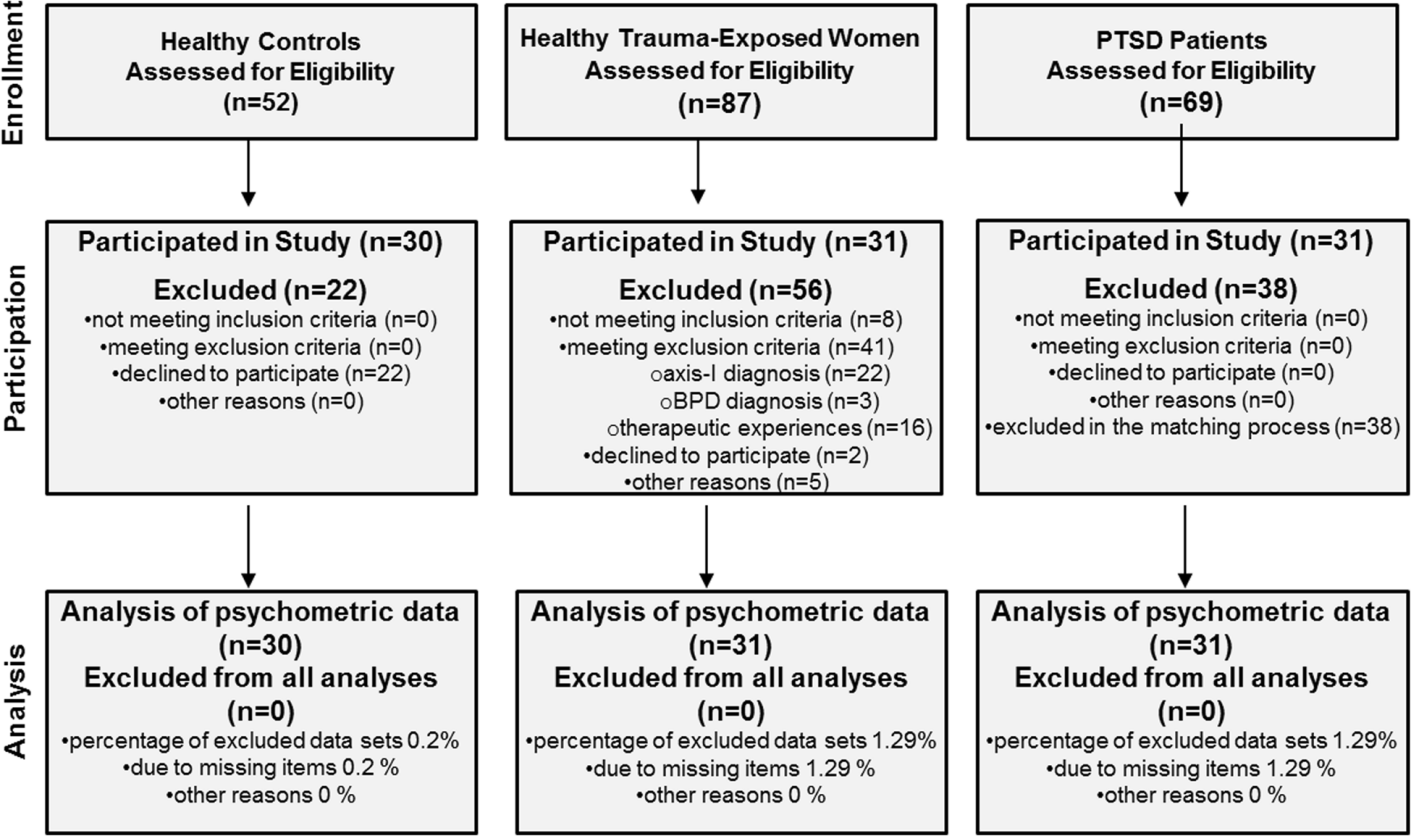
Participant flowchart

A total of 92 women participated in this study: 31 women with a history of potentially traumatic CSA/CPA and no axis-I diagnosis or BPD (HTEW), 31 patients meeting criteria for PTSD related to experiences of CSA/CPA, and 30 healthy controls without experiences of childhood interpersonal violence or other traumatic events (HC; see Fig. 1). Enrollment was restricted to women aged between 18 and 65. For the individuals of the HTEW group, inclusion criterion was the experience of sexual or physical abuse before the age of 18. Exclusion criteria were meeting criteria for a lifetime diagnosis of any axis-I disorder or of BPD, intake of psychotropic drugs or having received a psychotherapeutic intervention in the form of seeing a therapist or counsellor. The same exclusion criteria applied for the HC group plus experiences of interpersonal violence in childhood or adolescence or any other traumatic events that met the PTSD criterion A. For the patient sample, inclusion criterion was a current diagnosis of PTSD according to DSM-5 that was related to experiences of sexual or physical abuse before the age of 18. Experiences of sexual or physical abuse had to be the index trauma, meaning the most burdensome event, leading to PTSD. Because the PTSD patients who participated in our study also took part in a study comparing two different psychological treatments for PTSD and co-occurring BPD-features, they additionally had to fulfill at least three criteria of BPD (including criterion 6: affective instability) as defined by the International Personality Disorder Examination (IPDE; [37]). Seventeen out of 31 participants in our PTSD sample met full criteria for BPD. The additional inclusion criterion for the PTSD patient group, i.e. at least 3 out of 9 criteria of BPD was chosen as we wanted to include highly impaired patients with complex PTSD. Exclusion criteria for this group were a lifetime diagnosis of schizophrenia or bipolar-I disorder, current substance dependence, a body mass index <16 or intellectual disability as objectified by a verbal intelligence test [38]. For safety reasons, individuals who had attempted suicide within the last 2 months were also excluded. The diagnostic interviews to assess inclusion and exclusion criteria were conducted by trained clinical psychologists using the BPD section of the IPDE, the Structured Clinical Interview for DSM-IV (SCID-I; [39]) and the Clinician Administered PTSD Scale for DSM-5 (CAPS; [40]).

Individuals of the HTEW group were recruited through newspaper advertisements and flyers distributed at public places (e.g., cafés, supermarkets). The HC sample was recruited via the database at the Department for Psychosomatic Medicine and Psychotherapy, CIMH Mannheim, which contains contact information of pre-screened healthy controls. Data of PTSD patients were obtained from an ongoing multi-center study at the Department for Psychosomatic Medicine and Psychotherapy, CIMH Mannheim, of the Department of Clinical Psychology and Intervention, Institute of Psychology, Goethe University Frankfurt, and the Department of Psychology, Faculty of Life Sciences, Humboldt-Universitaet zu Berlin.

Because the central inclusion criterion for individuals of the HTEW and PTSD groups was the experience of CSA or CPA, 31 PTSD patients with a comparable level of physical and sexual abuse as the individuals in the HTEW group were taken out of the PTSD patient pool of the multi-center study. This was achieved by matching the scores of the two subscales of Sexual Abuse and Physical Abuse of the Childhood Trauma Questionnaire (see Table 2; CTQ; Bernstein & Fink, 1998) as implemented in a study by Roy et al. [41]. Furthermore, all three groups were matched for age (HC: 31.4 ± 8.4; HTEW: 31.8 ± 12.6; PTSD patients: 32.9 ± 8.7; *F*[2,89] = .189, *p =* .828) and years of education (HC: 11.3 ± 1.0; HTEW: 11.4 ± 0.9; PTSD patients: 10.9 ± 1.2; *H*[2] =3.74, *p* = .155). Approval was obtained from the independent Ethics Committee of the Medical Faculty Mannheim at Heidelberg University. All participants provided written informed consent.

### Procedure and measures

Severity of experiences of childhood interpersonal violence was assessed by the total score and subscale scores of the CTQ [36]. The CTQ is composed of 28 items subdivided into five subscales of adverse events (Physical Abuse, Physical Neglect, Sexual Abuse, Emotional Abuse, and Emotional Neglect) and a minimization/denial subscale. Participants rate the frequency of maltreatment for each item on a five-point Likert scale. The CTQ total score (all subscales except the minimization/denial scale) ranges from 25 to 125, and the five subscale scores of adverse events range from 5 to 25. In this study, individuals of the HTEW group and PTSD patients were matched with respect to the two subscales of Physical Abuse and Sexual Abuse. The matching procedure was restricted to these two subscale scores, because experiences of physical or sexual abuse were the central inclusion criterion of the study.

The assessment of PTSD-specific psychopathology is comprised of the *Davidson Trauma Scale (DTS;* [42]*),* the *Trauma Related Guilt Inventory (TRGI;* [43]*),* and the German adaption *(Fragebogen zu Dissoziativen Symptomen (*FDS*;* [44]*) of the Dissociative Experiences Scale* [45]*).*

**Fig. 2:**
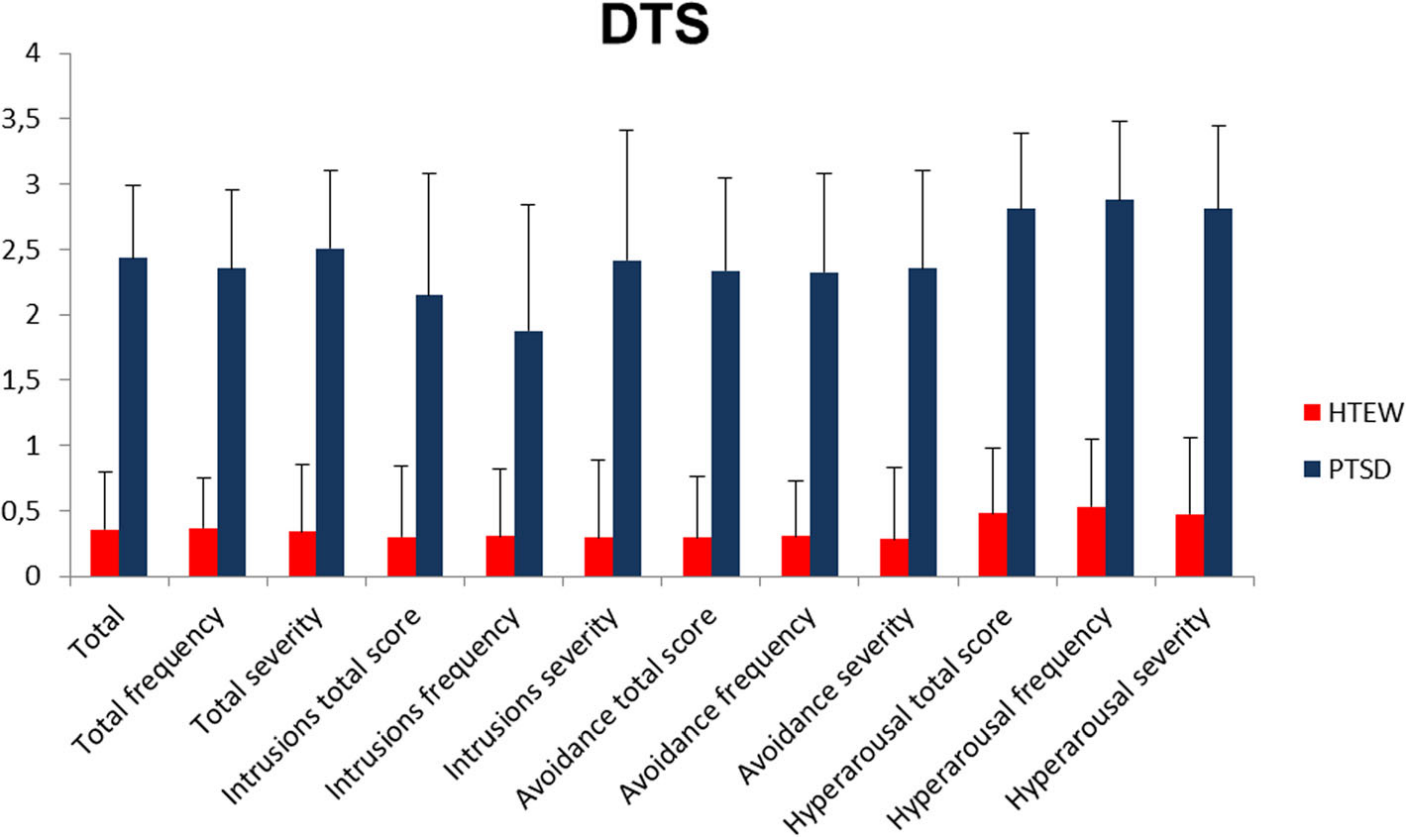
DTS total and subscale mean scores for individuals in the HTEW group and PTSD patients. Error bars are depicted as standard deviations (SD)

The DTS is a 17-item self-report questionnaire measuring each DSM-IV symptom of PTSD on five-point frequency and severity scales for a total possible score between 0 and 136. Subscale scores for intrusive re-experiencing, avoidance and numbness and hyperarousal can be computed separately for frequency and severity (see Fig. 2).

The TRGI is a valid 32-item measure of trauma-related guilt. Every item is rated on a five-point Likert scale. It implies the three scales of global guilt (4 items with a total score ranging from 0 to 4), distress (6 items with a total score ranging from 0 to 4), and guilt cognitions (21 items with a total score ranging from 0 to 4).

The FDS is an easy applicable, reliable, and valid measure to quantify dissociative experiences that is based on the Dissociative Experience Scale. It is comprised of 44 items rated on a 10-point scale with a total score ranging from 0 to 100.

The assessment of general psychopathology and psychological flexibility is comprised of the *Beck Depression Inventory-II* (BDI-II; [46])*,* the *Brief Symptom Inventory (*BSI; [47]*),* the *Global Assessment of Functioning (*GAF; [48]*),* the *Barratt Impulsiveness Scale Version 10 (*BIS*;* [49]*),* the *Acceptance and Action Questionnaire-II (*AAQ-II; [50]*),* and the *Rosenberg Self-Esteem Scale (*SES; [51]*)*.

The BDI-II is the most widely used self-report questionnaire worldwide to evaluate severity of depressive symptoms. It contains 21 items rated on a four-point Likert scale with a total score ranging from 0 to 63.

The BSI is a 53-item self-report symptom inventory designed to assess psychological distress and psychiatric disorders. All items are rated on a five-point Likert scale from 0 to 4 with a total score ranging from 0 to 4.

The GAF is a single rating scale for evaluating a person’s psychological, social, and occupational functioning on a hypothetical continuum of global level of functioning and ranges from 1 to 100.

The BIS is a 34-item self-report questionnaire to measure impulsiveness. All items are answered on a four-point Likert scale from 1 to 4.

The AAQ-II assesses experiential avoidance and psychological flexibility. Psychological flexibility is defined as the ability to fully contact the present moment with all its inherent thoughts and feelings. It implies to either persist in or change the behavior that is necessary in the pursuit of goals and values [52]. It is comprised of 7 items that are answered on a seven-point Likert scale ranging from 7 to 49, with higher summed scores indicating higher psychological inflexibility.

The SES is a self-rating instrument that assesses global self-esteem. It contains ten items that are rated on a four-point Likert scale ranging from 0 to 30. Scores ranging between 15 and 25 indicate normal self-esteem, while scores below 15 indicate low self-esteem.

Quality of Life and sexual satisfaction were assessed using the *Satisfaction with Life Scale (*SWLS; [53]*),* the *EQ-5D* [54]*,* the *WHOQOL-BREF* [55] and the *Resources in Sexuality and Partnership* (RSP; [56]*).*

The SWLS is a five-item scale that assesses general life satisfaction. Each item is rated on a seven-point scale with a total score ranging from 5 to 35. The higher the total score, the higher the level of experienced global life satisfaction is.

The EQ-5D is a short questionnaire for measuring health-related quality of life. It consists of 5 items that are rated on a three-point Likert scale with a total score ranging from 5 to 15. The total score is then transformed into a scale ranging from 0 to 100.

The WHOQOL-BREF is the most frequently used questionnaire to assess QoL and consists of 26 five-point Likert scale (1–5) items. It includes four main domains of QoL (physical health, psychological health, social relationships and environment) and a facet of overall QoL and general health. All five scores are transformed into a range from 0 to 100 to ensure comparability.

The RSP encompasses five domains assessing the subjective experience of joy with respect to physical attractiveness, tenderness, sexual lust and satisfaction, love and communication of sexual needs and desires. We used a modified version of the 25-item RSP and included a sixth response option, “did not happen,” because many of our patients do not have sexual intercourse. On the one hand, we assessed a total RSP score of all items that were answered from 1 (very rarely enjoyable) to 5 (very often enjoyable) to assess joy and satisfaction with sexuality. On the other hand, we assessed the percentage of items that were answered with “did not happen” to assess the incidence of sexual behaviors. The questionnaire can be applied independently of sexual orientation and kind of interpersonal relationship.

Resilience was assessed using the *Resilience Scale* [57], which measures resilience “as the ability … to use internal and external resources successfully to cope with developmental tasks” ([47], p. 21) on a 25-item scale. It is comprised of a total score and two subscale scores. The subscale Personal Competence subsumes characteristics such as self-confidence, autonomy, mastery, mobility and endurance. The subscale Acceptance of Self and Life subsumes characteristics such as adaptability, tolerance, flexible sight towards oneself and towards one’s journey through life. Items are rated on a seven-point Likert scale and summarized for each subscale.

### Statistical analysis

All analyses were performed using SPSS (version 21;SPSS Inc.;USA). To test for differences between the HTEW group, PTSD patients, and the HC group on the questionnaires and for age, one-way analyses of variance (ANOVAs) were applied. Post-hoc analyses were performed by pairwise comparisons (Bonferroni corrected for multiple testing). Non-parametric Kruskal-Wallis tests were used for overall comparisons in years of education, employment status, and number of children across the three groups. Pairwise post-hoc comparisons were conducted with Mann-Whitney U tests. To test for differences in marital status, chi-square tests were applied. Effect sizes were calculated as Cohen’s d. A significance level of *p* ≤ .05 (two-tailed) was applied for all analyses. Data were reported as arithmetic mean (AM) ± standard deviation (SD).

## Results

Demographic characteristics of the three groups (HC, HTEW, PTSD patients) are presented in Table 1. The three groups did not differ significantly in terms of age, education, marital status, and number of children, but did with regards to employment status. Herein, the HTEW and HC group did not differ significantly with 87.1 % of HTEW and 76.7 % of HC working full or part time. However, both groups differed significantly from PTSD patients of whom only 42 % were working full or part time.

**Table 1:** Sample characteristics of healthy controls, healthy trauma-exposed women and PTSD patients

	*HC*		*HTEW*		*PTSD* *patients*		*Statistics*
	AM ± SD		AM ± SD		AM ± SD		
Age	31.37 ± 8.4		31.81 ± 12.6		32.9 ± 8.6		*F*(2,89) = .189, *p = .828*
Years of education	11.3 ± 1.0		11.4 ± 0.9		10.9 ± 1.2		*H* (2) = 3.735, *p =* .155
Marital status	*N*, %		*N*, %		*N*, %		
Married/cohabiting Single Divorced/widowed	24 (80.0) 6 (20.0) 0 (0)		20 (64.5) 8 (25.8) 3 (9.7)		19 (61.3) 11 (35.5) 1 (3.2)		*χ* ^2^ (2) = 4.645, *p =* .336
Number of children	*N*, %		*N*, %		*N*, %		
1 2 3 4	22 (73.4) 3 (10.0) 3 (10.0) 1 (3.3) 1 (3.3)		21 (67.7) 2 (6.5) 2 (6.5) 5 (16.1) 1 (3.2)		19 (61.3) 6 (19.4) 4 (12.9) 1 (3.2) 1 (3.2)		*H* (2) = .774, *p* = .*679* *U*_HC-HTEW_ = 427.0, *p =* .489*,* r = −.087 *U*_HTEW-PTSD_ = 475.5, *p =* .941*,* r = −.011 *U*_HTEW-PTSD_ = 416.0, *p =* .402, r = −.109
Employment status	*N*, %		*N*, %		*N*, %		
Full time Part time Occasionally Not working	16 (53.4) 7 (23.3) 4 (13.3) 3 (10.0)		16 (51.6) 11 (35.5) 3 (9.7) 1 (3.2)		7 (22.6) 6 (19.4) 3 (9.7) 15 (48.4)		*H* (2) = 14.451, *p =* .001 *U*_HC-HTEW_ = 446.0, *p* = .769*,* r = −.039 *U*_HTEW-PTSD_ = 246.5, *p < .001,* r = .439 *U*_HC-PTSD_ = 266.0, *p < .001.*, r = −.387
Current comorbidity	*N*	%	*N*	%	*N*	%	–
Any anxiety disorder	0	0	0	0	22	71.0	–
Any mood disorder	0	0	0	0	21	67.7	–
Any eating disorder	0	0	0	0	4	12.9	–
Any substance abuse/dependence	0	0	0	0	1	3.2	–
BPD	0	0	0	0	17	54.8	

F and p represent the F- and *p*-values of the respective one-way analysis of variance (ANOVA); H and U represent the respective H and U values of the Kruskal-Wallis and Mann-Whitney-U tests; *χ* ^2^ represents the respective value of the Chi-square analysis

*Abbreviations*: *AM* arithmetic mean, *SD* standard deviation

For Axis-II Disorders, only Borderline Personality Disorder was assessed

## Experiences of childhood interpersonal violence

With regards to the CTQ total score, one-way ANOVA yielded significant differences between the three groups (*F(*2,87) = 68.798, *p* < .001). Post-hoc pairwise comparisons showed that PTSD patients reported a significantly higher frequency of adverse events compared to individuals of the HTEW group (*p* < .001), who in turn reported a higher frequency of adverse events than HC (*p* < .001). As Table 2 shows, the frequency of adverse events in childhood varied between groups within the different subscales: PTSD patients and participants within the HTEW group did not differ significantly concerning sexual (*p* = 1.0) and physical abuse (*p* = .94) as a consequence of our matching procedure. However, both groups differed significantly from individuals in the HC group within these subscales (*p* < .001). With regard to the subscale of physical neglect, PTSD patients experienced a significantly higher frequency of physical neglect compared to participants in the HTEW and HC groups (*p* < .001), whereas participants in HTEW and HC groups did not differ significantly (*p* = .227). Concerning emotional abuse and emotional neglect, PTSD patients experienced a significantly higher frequency of adverse events than individuals in the HTEW group, who in turn reported a significantly higher frequency than individuals in the HC group (*p* < .001).

**Table 2:** Childhood Trauma Questionnaire total and subscale scores of healthy controls, healthy trauma-exposed women and PTSD patients

	*HC*	*HTEW*	*PTSD* *patients*	*Statistics*					
	AM ± SD	AM ± SD	AM ± SD	F	df	*p*	d _HTEW-HC_	d _HTEW-PTSD_	d _PTSD-HC_
CTQ-total score	31.8 ± 7.8	52.9 ± 13.4	69.9 ± 15.3	68.798	(2,87)	< .001	1.99	−1.18	3.3
CTQ-Sexual Abuse	5.1 ± 0.3	10.9 ± 5.8	11.6 ± 6.0	16.579	(2,87)	< .001	1.87	−.12	2.06
CTQ-Physical Abuse	5.9 ± 2.4	9.5 ± 3.9	10.2 ± 4.6	11.06	(2,87)	< .001	1.14	−.16	1.23
CTQ-Emotional Abuse	7.1 ± 2.8	12.7 ± 5.1	18.2 ± 5.5	43.489	(2,87)	< .001	1.42	−1.04	2.67
CTQ-Emotional Neglect	7.8 ± 3.4	12.5 ± 4.5	18.8 ± 4.4	53.621	(2,87)	< .001	1.19	−1.42	2.82
CTQ-Physical Neglect	6.0 ± 1.9	7.2 ± 2.5	8.1 ± 3.4	30.055	(2,87)	< .001	0.55	−0.31	0.79

F and p represent the F- and *p*-values of the respective one-way analysis of variance (ANOVA); d represents the pairwise compared effect sizes after Cohen

*Abbreviations*: *df* degrees of freedom, *AM* arithmetic mean, *SD*, standard deviation, *CTQ* Childhood Trauma Questionnaire

## PTSD-specific and general psychopathology

Not surprisingly, PTSD patients reported significantly more PTSD-specific symptoms than individuals in the HTEW group in general. Examining PTSD-specific symptoms within the scope of the DTS, Fig. 2 illustrates frequency and severity mean scores for the three symptom clusters of the diagnostic criteria (intrusive experiences, avoidance of trauma related triggers, hyperarousal) separately. For the frequency subscales, PTSD patients reported a significantly higher frequency of experienced symptoms such as intrusions, avoidance of trauma related triggers, and hyperarousal compared to HTEW participants. The same pattern was observed for severity scores on all subscales, with PTSD patients reporting symptoms as significantly more severe than the HTEW participants (see Fig. 2).

With regard to general psychopathology, comparisons of the three groups revealed a similar pattern in all questionnaires. PTSD patients showed significantly higher levels of psychopathology compared to the HTEW and HC groups, respectively, in terms of depression (BDI-II), psychological distress and psychiatric disorders (BSI), global functioning (GAF), impulsivity (BIS), psychological flexibility (AAQ-II), self-esteem (SES), trauma-related guilt cognitions (TRGI), and dissociative symptoms (FDS). For detailed information, see Table 3. Within all those measures, individuals in the HTEW group showed a very high level of functioning (e.g., GAF:87) and a very low level of psychopathological impairment (e.g., BSI:0.3; BDI-II:4.5), which were comparable to levels of individuals in the HC group. Effect sizes suggest that differences between HTEW participants and PTSD patients were highest in the GAF and the AAQ-II (GAF: d = 5.95; AAQ-II: d = −4.72). The same pattern occurred between PTSD patients and the HC group (GAF: d = −5.77; AAQ-II: d = 5.49). The highest differences between participants in the HTEW and HC groups were found in the FDS (d = 0.6). The effect sizes for all psychopathological measures between HTEW participants and PTSD patients ranged between −1.58 and 5.05, and for all QoL measures, between 1.57 and 3.94. Comparing individuals in the HTEW and HC groups with regard to psychopathological measures, effect sizes ranged between −0.02 and 0.6, and for QoL, between −0.01 and −0.34.

**Table 3:** General and PTSD-specific psychopathology of healthy controls, healthy trauma-exposed women and PTSD patients

	*HC*	*HTEW*	*PTSD* *patients*	*Statistics*					
	AM ± SD	AM ± SD	AM ± SD	F	df	*p*	d _HTEW-HC_	d _HTEW-PTSD_	d _PTSD-HC_
BDI-II	4.6 ± 4.7	4.5 ± 6.0	38.6 ± 10.1	222.268	(2,89)	< .001	−.02	−4.24	4,57
BSI	0.2 ± 0.2	0.3 ± 0.3	2.0 ± 0.6	206.837	(2,88)	< .001	0.4	−3.78	4.46
GAF	91.1 ± 8.3	86.6 ± 8.6	48.5 ± 6.5	273.92	(2,89)	< .001	−0.53	5.05	−5.77
BIS	60.3 ± 9.2	64.3 ± 9.3	83.0 ± 14.3	35.91	(2,89)	< .001	0.43	−1.58	1.92
AAQ-II	12.2 ± 5.7	13.9 ± 6.6	39.6 ± 4.3	231.167	(2,89)	< .001	0.28	−4.72	5.49
SES	27 ± 4.1	26.9 ± 4.0	9.3 ± 4.8	174.51	(2,88)	< .001	−0.02	3.99	−3.97
*DTS*	–	12.2 ± 14.8	82.6 ± 19.1	264.81	(1,60)	< .001	−	−4.15	−
TRGI-global guilt	–	0.6 ± 0.8	2.7 ± 1.2	61.570	(1,55)	< .001	–	−2.12	–
TRGI-distress	–	1.0±0.8	3.5 ± 0.4	198.623	(1,55)	< .001	–	−4.09	–
TRGI-guilt cognitions	–	1.0±0.5	2.1 ± 0.6	46.202	(1,55)	< .001	–	−2.01	–
FDS	2.8 ± 2.4	5.0 ± 5.0	24.4 ± 12.2	69.402	(2,86)	< .001	0.6	−2.21	2.93

F and p represent the F- and *p*-values of the respective one-way analysis of variance (ANOVA); d represents the pairwise compared effect sizes after Cohen

*Abbreviations*: *df* degrees of freedom, *AM* arithmetic mean, *SD* standard deviation, *BDI-II* Beck Depression Inventory-II, *BSI* Brief Symptom Inventory, *GAF* Global Assessment of Functioning, *BIS* Barratt Impulsiveness Scale, *AAQ* Acceptance and Action Questionnaire, *SES* Rosenberg Self Esteem Scale, *DTS* Davidson Trauma Scale, *TRGI* Trauma Related Guilt Inventory, *FDS* Fragebogen zu Dissoziativen Symptomen, *AM* arithmetic mean, *SD* standard deviation

Dashes indicate that data were not obtained

## Quality of life and sexual satisfaction

**Fig. 3:**
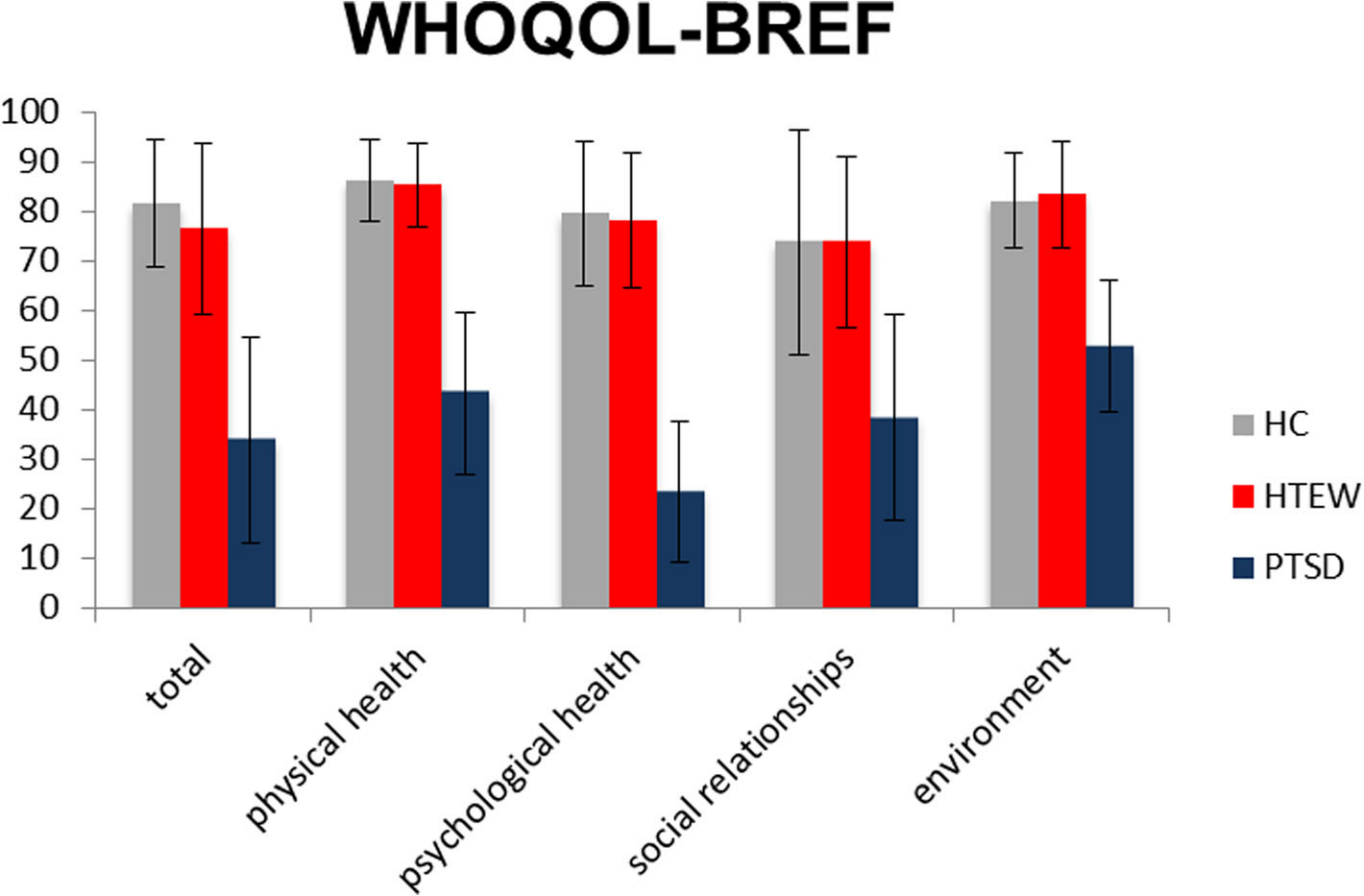
WHOQOL-BREF total and subscale scores for all three groups. Error bars are depicted as standard deviations (SD)

Analysis of all three QoL measures (WHOQOL-BREF, SWLS, EQ-5D) showed that the groups differed significantly in subjectively experienced QoL (*F*_SWLS_(2, 89) = 101.206, *p* < .001; *F*_EQ-5D_(2, 88) = 93.284, *p* < .001; *F*_WHOQOL-BREF global_(2, 88) = 69.355, *p* < .001). Post-hoc pairwise comparisons of all three measures indicated that both HTEW and HC participants reported higher QoL levels compared to PTSD patients (*p* < .001), whereas HTEW participants showed QoL levels that were comparable to HC participants (*p* = 1.0; d_SWLS_ = 0.23; d_EQ5D_ = −0.27; d_WHOQOL-BREF_ = −0.34). This pattern also emerged within the WHOQOL-BREF subscales of satisfaction with physical health, psychological health, social relationships, and the environment (see Table 4 and Fig. 3).

**Table 4:** Quality of life, sexual satisfaction and resilience of healthy controls, healthy trauma-exposed women and PTSD patients

	*HC*	*HTEW*	*PTSD patients*	*Statistics*					
	AM ± SD	AM ± SD	AM ± SD	F	df	*p*	d _HTEW-HC_	d _HTEW-PTSD_	d _PTSD-HC_
SWLS	28.3 ± 4.7	27.1 ± 5.7	10.8 ± 5.6	101.206	(2,89)	< .001	0.23	2.88	−3.39
EQ-5D	97 ± 5.3	95.5 ± 5.7	66.3 ± 15.2	93.284	(2,88)	< .001	−0.27	2.82	−3.0
WHOQOL-BREF-global	81.5 ± 12.8	76.4 ± 17.4	33.9 ± 20.7	69.355	(2,88)	< .001	−0.34	2.23	−3.58
WHOQOL-physical health	86.2 ± 8.1	85.3 ± 8.3	43.3 ± 16.3	136.433	(2,88)	< .001	−0.11	3.41	−3.48
WHOQOL-psychological health	79.6 ± 14.5	78.2 ± 13.7	23.4 ± 14.1	158.372	(2,88)	< .001	−0.1	3.94	−3.93
WHOQOL-social relationships	73.9 ± 22.6	73.8 ± 17.3	38.4 ± 20.7	31.208	(2,88)	< .001	−0.01	1.86	−1.64
WHOQOL-environment	82.1 ± 9.6	83.5 ± 10.7	52.8 ± 13.3	71.948	(2,88)	< .001	0.14	2,56	−2.54
RSP-items indicating no sexual activity (in %)	10.5 % ± 17.3 %	9.4 % ± 13.9 %	45.8 % ± 35.0 %	22.979	(2,89)	< .001	−0.07	−1.49	1.35
RSP	82.8 ± 28.4	84.8 ± 24.2	39.5 ± 33.4	24.114	(2,89)	< .001	0.08	1.57	−1.40
RS	146.27 ± 14.9	144.4 ± 14.6	83.4 ± 20.6	137.022	(2,89)	< .001	−0.13	3.47	−3.53
RS-personal competence	101.6 ± 9.7	101.1 ± 9.2	61.6 ± 16.3	109.370	(2,89)	< .001	−0.05	3.1	−3.06
RS-acceptance of self & life	44.7 ± 6.9	43.3 ± 6.6	21.8 ± 5.5	124.041	(2,89)	< .001	−0.21	3.55	−3.7

F and p represent the F- and *p*-values of the respective one-way analysis of variance (ANOVA); d represents the pairwise compared effect sizes after Cohen

*Abbreviations*: *df* degrees of freedom, *AM* arithmetic mean, *SD* standard deviation, *SWLS* Satisfaction with Life Scale, *EQ-5D* Quality of life measure of the EuroQol Group, *WHOQOL-BREF* World Health Organization Quality of Life BREF Version, *RSP* Resources in Sexuality and Partnership, *RS* Resilience Scale

**Fig. 4:**
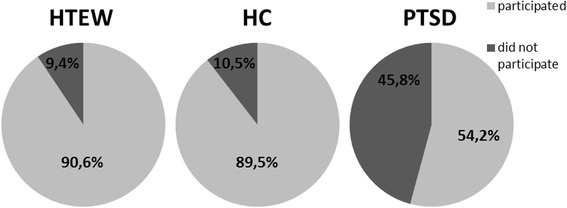
Percentage of items indicating participation in sexual activities with partners, and feeling more sexually satisfied and attractive within the last 4 weeks

Examining the incidence of sexual behaviors, analyses of the RSP indicated that both individuals in the HTEW and HC groups participated significantly more often in sexual activities with partners, experienced more sexual satisfaction, and had felt more attractive in the last 4 weeks compared to PTSD patients (*F*(2, 89) = 22.979, *p* < .001; see Table 4 and Fig. 4).

Concerning sexual satisfaction, individuals of the HTEW and HC groups were significantly more satisfied with their sexuality compared to PTSD patients (*F*(2, 89) = 24.114, *p* < .001; see Table 4). In a second step, median split analyses were conducted for the PTSD group to investigate separately the satisfaction scores of PTSD patients for sexually active versus sexually inactive participants. The PTSD patient group was subdivided by median split into two groups: PTSD patients scoring more items than median with “did not happen,” referred to as low_sexually_active versus PTSD patients scoring fewer items than median with “did not happen,” referred to as high_sexually_active. Whereas in the analyses of sexual satisfaction of the three groups (HTEW vs. HC vs. PTSD patients) the PTSD patients reported significantly less sexual satisfaction, this pattern changed when PTSD patients were split into two groups (low_sexually_active vs. high_sexually_active). Post-hoc pairwise comparisons showed that individuals in the HTEW group, individuals in the HC group, and high_sexually_active PTSD patients (AM = 67.33) did not differ significantly in sexual satisfaction (*p*_HTEW-high_sexually_active_PTSD_ = .147, d_HTEW-HighPTSD_ = 0.72; *p*_HC-high_sexually_active_PTSD_ = .287, d_HC-HighPTSD_ = 0.57) and were comparably satisfied, whereas low_sexually_active PTSD patients (AM = 13.50) did differ significantly from all other groups (*p* < .001) with significantly lower satisfaction scores (RSP_HTEW_:84.82_;_ RSP_HC_:82.77_;_ RSP_high_sexually_active_PTSD_:67.33; RSP_low_sexually_active_PTSD_:13.50).

## Resilience

Individuals in both the HTEW and HC groups reported significantly higher resilience scores compared to PTSD patients. This pattern was observed for the total score as well as the two subscale scores (*F*_total_(2,89) = 137.022, *p* ≤ .001; *F*_personal competence_(2,89) = 109.370, *p* ≤ .001; *F*_acceptance_(2,89) = 124.041, *p* ≤ .001; see Table 4).

## Discussion

This study investigated subthreshold symptoms of PTSD-specific and general psychopathology and impairments in global functioning, quality of life, and sexuality in women with a history of potentially traumatic CSA/CPA without current or lifetime axis-I disorders or BPD. Results of this particular group were compared to those of women with PTSD related to a history of potentially traumatic CSA/CPA and potentially other axis-I disorders and healthy control women without traumatic experiences. Overall, we did not find any psychopathology in individuals in the HTEW group, who did not show subthreshold psychopathologic symptoms or exhibit unspecific restrictions in psychological well-being. Additionally, no measurable restrictions in quality of life and sexual satisfaction were observed. They showed a high level of functioning (e.g., GAF:87 and 51.6 % are working full time) and a low level of psychopathology (e.g., BSI: 0.3; BDI-II:4.5), which was comparable to those levels in the HC group.

These findings confirm that psychopathology is not an inevitable consequence of traumatic experiences. Prospective longitudinal studies and large epidemiologic studies have shown that the conditional probability of developing PTSD range between 12 and 20 % [3, 4] and that many individuals affected by PTE did not seek psychotherapeutic help in the aftermath of PTE [9]. Our findings suggest that traumatic experiences such as CSA or CPA per se do not explain the development of psychopathology in the aftermath of PTE. It is more likely that an interaction of different factors such as perceived support from others following trauma, health status at time of trauma, psychopathology in family of origin, psychological problems prior to trauma, peritraumatic dissociation, cognitive abilities, and personality factors plays a role in the process of overcoming traumatic experiences [58, 59].

Several possible explanations could explain the differences occurring in individuals in the HTEW group versus PTSD patients in our study. While both groups experienced childhood physical or sexual abuse, differences between the groups may be related to the experience of emotional abuse. In our study, PTSD patients experienced a significantly higher frequency of emotional abuse and emotional neglect compared to HTEW participants. This would be in line with findings by Nash et al. [60] stating that pathological family environments account for psychological impairments rather than does the experience of sexual abuse. A similar pattern was observed by Corso et al. [61] concerning the impact of emotional abuse on QoL. Here, previous research shows that experiences of emotional neglect had the strongest impact on perceived QoL followed by sexual abuse and physical abuse. Contradicting these findings, Lewis et al. found that children with a history of CSA had significantly more behavioral problems with greater externalizing and internalizing problems compared to children, who were maltreated but not sexually abused [62]. However, in our study, individuals in the HTEW group experienced a significantly higher frequency of emotional abuse and emotional neglect compared to individuals in the HC group and did not differ in psychopathology and QoL.

Furthermore, the traumatized but healthy participants in our study reported almost no guilt cognitions concerning the traumatic event, whereas PTSD patients reported moderate to intense guilt cognitions. Our findings are in line with other studies that found that not the trauma per se but rather the meanings of traumatic events are important [58, 59]. Guilt is defined as a belief that one should have thought, felt or acted differently [63], so to speak, an evaluation of one’s own behavior as failure. In the context of experiences of interpersonal violence, guilt cognitions are usually about not having defended oneself enough or having deserved what had happened [64].

The result that individuals in the HC and HTEW groups have almost identical scores on the resilience scale is surprising, given the fact that overcoming a history of CSA or CPA without any indicators of psychopathological symptoms can be referred to as being resilient according to most definitions of resilience (“relatively positive psychological outcomes,”, p.1; [65, 66]). Accordingly, the individuals in the HTEW group should have values on any resilience scale that exceed general population norm values and differ from a healthy population. One could argue that individuals in the HTEW group were resilient at a different time of their lives, namely, the years after the trauma, and that their score has declined in the process of coping with the trauma. Considering that the Resilience Scale was designed to measure resilience in adulthood, it seems more likely that other protective factors helped these individuals in childhood or adolescence to be resilient and survive in good psychological health (for protective factors in children, see [67]). There is an increasing discussion on the operationalization of resilience as a personality pattern or bundle of protective factors [68].

Some limitations of the study have to be considered. A first limitation relates to the representative state of our HTEW group for this population. In our study, the healthy subgroup of participants with a history of potentially traumatic CSA and CPA (HTEW) was highly selective due to the restrictive inclusion criteria. None of the individuals in the HTEW group in this study fulfilled the criteria of any mental axis-I disorder or BPD, never attended psychotherapy sessions, and never took psychotropic drugs. This group seemed to subjectively and objectively get along with what happened and decided to voluntarily participate in this study and communicate about what had happened to them. We chose this healthy subsample to prevent the data in the HTEW group from being affected by ramifications of other psychopathologies such as depression or anxiety disorders. This procedure strengthens our internal validity at an expense of external validity. Also, this limitation relates to the representative state of our PTSD sample. The PTSD sample in our study comprised highly impaired participants that would possibly meet the criteria for complex PTSD that comprises elevated PTSD symptoms as well as affective dysregulation. Second, all trauma data were obtained by (retrospective) self-report. Therefore, on the one hand, a participant’s recollection of CSA and CPA could have been influenced by recall bias. There is controversial evidence concerning accuracy of retrospective self-report of childhood adverse events. For example, Fergusson et al. showed that claims about limitations of retrospective reports of CSA/CPA may have been overstated and that well collected retrospective data may provide valid information [69]. Contradicting this finding, a recently published study by Mills et al. found a disparity between the incidences of CSA when measured by retrospective self-report or prospective government agency notification [70]. On the other hand we do not know for sure, whether the reported psychometric data represent a relatively stable condition or might have been biased by current psychological distress. Third, the design of this study was cross-sectional. Thus, we cannot ascertain any cause and effect relationship between traumatic experiences in childhood or adulthood and actual psychopathological characteristics, resilience scores, and satisfaction with quality of life and sexuality.

Our study has at least one implication for future research. In our study, we quantitatively examined the dimensional distribution of psychopathology in individuals in the HTEW group. A very interesting future study could conduct qualitative interviews with healthy participants affected by potentially traumatic CSA and CPA to find out what helped them overcome these potentially traumatic experiences in such a resilient way without developing any mental disorders.

## Conclusions

The present study showed that participants with a history of potentially traumatic childhood interpersonal violence without axis-I disorder or BPD show a high level of functioning and a very low level of pathological impairment that is comparable to the level of healthy controls. The contribution of this study relates to characterizing healthy participants affected by potentially traumatic CSA/CPA with regard to psychopathology. The findings of this study confirm earlier findings that traumatic experiences per se do not necessarily go along with the development of psychopathology or impaired quality of life, sexuality, self-esteem or guilt cognitions. Further studies are needed to determine what helps individuals in the aftermath of PTE to turn out in a resilient way.

## Abbreviations

AAQ-II: Acceptance and Action Questionnaire-II
AM: Arithmetic mean
ANOVA: Analysis of variance
BDI-II: Beck Depression Inventory II
BIS: Barratt Impulsiveness Scale
BPD: Borderline Personality Disorder
BSI: Brief Symptom Inventory
CAPS: Clinician Administered PTSD Scale
CIMH: Central Institute of Mental Health
CPA: Childhood physical abuse
CSA: Childhood sexual abuse
CTQ: Childhood Trauma Questionnaire
DSM: Diagnostic and Statistical Manual of Mental Disorders
DTS: Davidson Trauma Scale
FDS: Fragebogen zu Dissoziativen Symptomen
GAF: Global Assessment of Functioning
HC: Healthy controls
HTEW: Healthy trauma-exposed women
IPDE: International Personality Disorder Examination
OR: Odds ratio
PTE: Potentially traumatic event
PTSD: Posttraumatic Stress Disorder
QoL: Quality of life
RS: Resilience Scale
RSP: Resources in Sexuality and Partnership
SCID: Structured Clinical Interview for DSM-IV
SD: Standard deviation
SES: Self-esteem Scale
SWLS: Satisfaction with life scale
TRGI: Trauma Related Guilt Inventory
